# Behr syndrome and hypertrophic cardiomyopathy in a family with a novel *UCHL1* deletion

**DOI:** 10.1007/s00415-020-10059-3

**Published:** 2020-07-12

**Authors:** Grace McMacken, Hanns Lochmüller, Boglarka Bansagi, Angela Pyle, Angela Lochmüller, Patrick F. Chinnery, Steve Laurie, Sergi Beltran, Leslie Matalonga, Rita Horvath

**Affiliations:** 1 Department of Neurosciences, Royal Victoria Hospital, Belfast, UK; 2 Department of Neuropediatrics and Muscle Disorders, Faculty of Medicine, Medical Center – University of Freiburg, Freiburg, Germany; 3 Centro Nacional de Análisis Genómico (CNAG-CRG), Center for Genomic Regulation, Barcelona Institute of Science and Technology (BIST), Barcelona, Catalonia Spain; 4 Division of Neurology, Department of Medicine, Children’s Hospital of Eastern Ontario Research Institute, The Ottawa Hospital and Brain and Mind Research Institute, University of Ottawa, Ottawa, Canada; 5 Wellcome Centre for Mitochondrial Research, Translational and Clinical Research Institute, Newcastle University, Newcastle upon Tyne, UK; 6 GKT School of Medical Education, King’s College London, London, UK; 7 Department of Clinical Neurosciences, University of Cambridge School of Clinical Medicine, Cambridge Biomedical Campus, Cambridge, UK; 8 MRC Mitochondrial Biology Unit, University of Cambridge, Cambridge, UK; 9 Department of Clinical Neurosciences, University of Cambridge School of Clinical Medicine, John Van Geest Cambridge Centre for Brain Repair, Robinson Way, Cambridge, CB2 0PY UK

**Keywords:** Neurogenetics, Behr syndrome, Hereditary spastic paraplegia, Ataxia, Whole exome sequencing

## Abstract

**Background:**

Behr syndrome is a clinically distinct, but genetically heterogeneous disorder characterized by optic atrophy, progressive spastic paraparesis, and motor neuropathy often associated with ataxia. The molecular diagnosis is based on gene panel testing or whole-exome/genome sequencing.

**Methods:**

Here, we report the clinical presentation of two siblings with a novel genetic form of Behr syndrome. We performed whole-exome sequencing in the two patients and their mother.

**Results:**

Both patients had a childhood-onset, slowly progressive disease resembling Behr syndrome, starting with visual impairment, followed by progressive spasticity, weakness, and atrophy of the lower legs and ataxia. They also developed scoliosis, leading to respiratory problems. In their late 30’s, both siblings developed a hypertrophic cardiomyopathy and died of sudden cardiac death at age 43 and 40, respectively. Whole-exome sequencing identified the novel homozygous c.627_629del; p.(Gly210del) deletion in *UCHL1*.

**Conclusions:**

The presentation of our patients raises the possibility that hypertrophic cardiomyopathy may be an additional feature of the clinical syndrome associated with *UCHL1* mutations, and highlights the importance of cardiac follow-up and treatment in neurodegenerative disease associated with *UCHL1* mutations.

## Introduction

Hereditary spastic paraplegias (HSP) are a clinically and genetically diverse group of disorders characterized by progressive lower limb spasticity. Inheritance can be dominant, recessive or X-linked, or rarely mitochondrial [1]. Additional neurological manifestations include optic atrophy, cerebellar ataxia, and peripheral neuropathy [2]. SPG79 is caused by homozygous or compound heterozygous mutations in *UCHL1*, which encodes Ubiquitin C‐terminal hydrolase‐L1. In addition to spasticity, ataxia, and peripheral neuropathy, SPG79 is characterized by severe optic atrophy in the first decade of life. To date, mutations in *UCHL1* have been described in only three families [3–5].

Using whole exome sequencing, we have identified a further family with SPG79, carrying a novel homozygous deletion in *UCHL1*. Both siblings presented in childhood with motor developmental delay, optic atrophy leading to progressive visual loss, cerebellar ataxia, spastic paraparesis, and motor neuropathy. Intriguingly, both siblings also developed hypertrophic cardiomyopathy, a potentially modifiable clinical feature which has not been described in association with SPG79 before.

## Patients and methods

Patient 1 (P1) and Patient 2 (P2) were born to British parents from the North of England. There is no information on consanguinity. P1 was the product of a normal pregnancy and was born at full-term, and had normal early development. She first presented aged 8 years with visual problems and optic atrophy was detected. Her motor function gradually deteriorated over the next years and she developed progressive lower limb spasticity, foot deformities, and severe scoliosis requiring spinal surgery aged 16 years. Cognitive function was normal. She became non-ambulant following a femoral fracture aged 38 years. From the age of 39, she developed nocturnal hypoventilation and was commenced on overnight non-invasive ventilation.

**Fig. 1:**
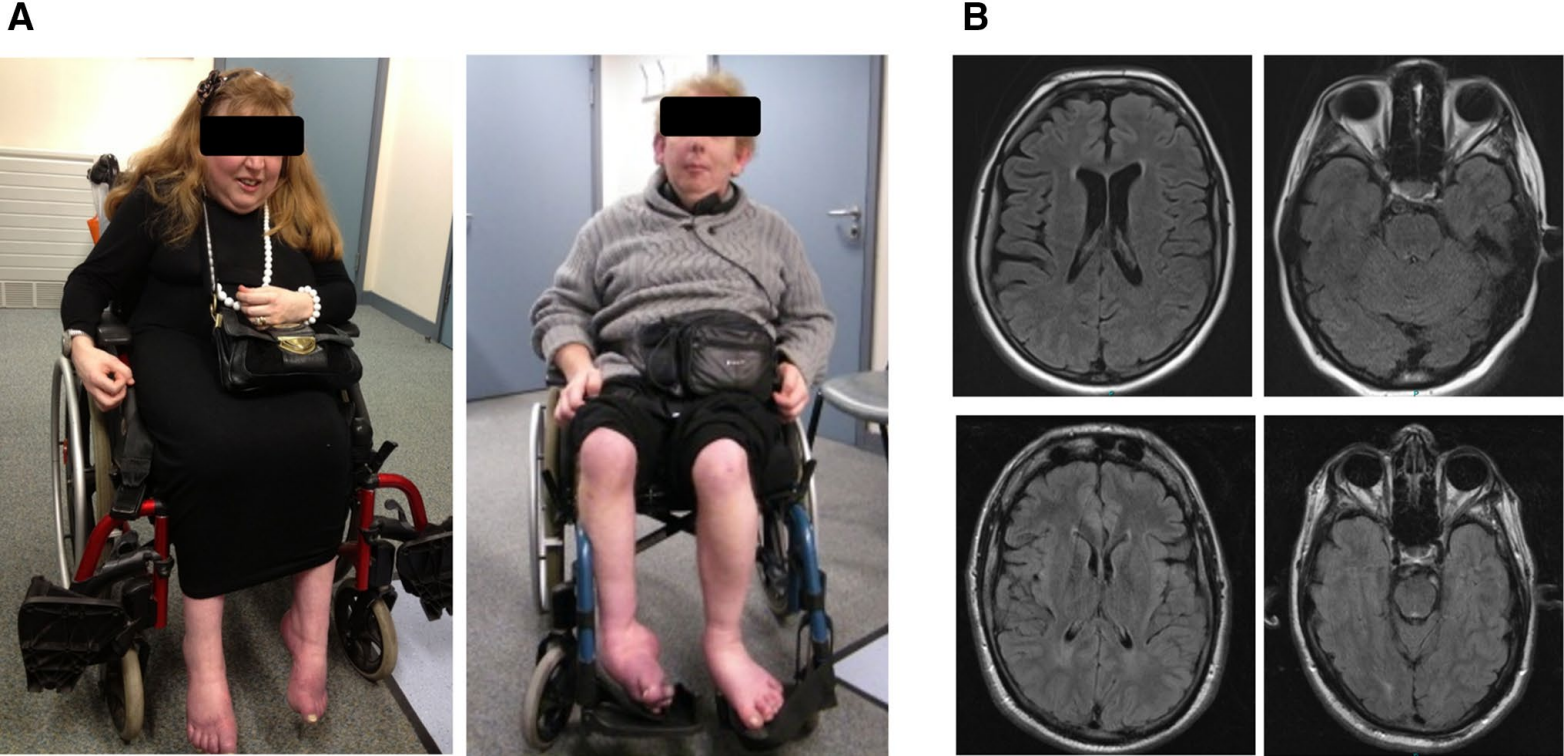
**a** P1 aged 40 years (left) and P2 aged 38 years. Both patients have characteristic facial features low-set ears and, and presented with distal upper and lower limb wasting and equinovarus deformity due to peripheral neuropathy and spastic paraparesis. **b** Axial T2 FLAIR MR images of P1 (top panels) and P2 (bottom panels). MRI brain in P1 aged 41 years showed mild global atrophy. MRI brain in P2 aged 34 demonstrated no significant abnormality apart from mild increased T2 signal in the peritrigonal white matter bilaterally

On examination aged 41 years (Fig. 1a), she had facial dysmorphism including low nasal bridge, micrognathia, and large, low-set ears, bilateral optic atrophy and severely reduced visual acuity, but no ophthalmoparesis or nystagmus. Deep tendon reflexes were absent in the upper and lower limbs, and there was length-dependent atrophy and sensory loss in-keeping with a peripheral neuropathy. She had marked lower limb spasticity, extensor plantar responses and ankle clonus, and a severe scoliosis.

P2, her younger brother, also presented in early childhood with reduced visual acuity. He developed lower limb spasticity, contractures, and foot deformity. He became non-ambulant aged 8 years following surgical correction of his foot deformity. His visual acuity continued to deteriorate due to optic atrophy, so that he was functionally blind. Cognitive function was also unaffected in P2.

Examination of P2 aged 33 (Fig. 1a) revealed bilateral optic atrophy, both pendular and gaze induced nystagmus, ophthalmoparesis, facial dysmorphism including low nasal bridge, micrognathia and large, low-set ears, and facial hypotonia. Deep tendon reflexes were pathologically reduced in upper and lower limbs, and plantar responses were extensor. Sensation was intact. He had severe finger–nose ataxia, dysdiadochokinesia, and truncal ataxia.

Detailed laboratory work-up (including acylcarnitines, urinary organic acids, very long chain fatty acids, pristanic and phytanic acids, lysosomal enzymes, copper, caeruloplasmin, and acanthocytes) was normal. CSF constituents were normal in both cases. MRI brain in P1 aged 41 years showed mild global atrophy and a small focus of non-specific periventricular T2 signal change at the right frontal horn (Fig. 1b). MRI brain in P2 aged 34 (Fig. 1b) demonstrated no significant abnormality apart from mild increased T2 signal in the peritrigonal white matter bilaterally. Unfortunately, neurophysiological testing was not technically possible due to the degree of foot deformity and swelling. Direct sequencing of *FXN* revealed no candidate pathogenic variants. Fifty-six gene inherited peripheral neuropathy panel (Bristol genetics laboratory) which also failed to identify any pathogenic variants and a comparative genomic hybridization (CGH) array was normal.

Given the symptomatic features of a possible mitochondrial disorder, in particular optic atrophy, a cardiac evaluation was carried out in both patients. Echocardiogram in P2 aged 33 years revealed a small, globally hypertrophied left-ventricular cavity, left-ventricular systolic ejection gradient (36 mmHg), and impaired diastolic left-ventricular function. Echocardiogram in P1 aged 41 years was technically difficult due to severe scoliosis, but also demonstrated cardiomyopathy, with moderately impaired left-ventricular function. The left-ventricular posterior wall was hypokinetic and the interventricular septum was dyskinetic. There was no significant family history of sudden cardiac death, arrhythmias, heart failure, or young stroke.

Both siblings were commenced on ACE inhibitors and beta-blockers and their cardiac function stabilized. Despite this, both siblings developed progressive shortness of breath and apnoeic episodes. P1 died aged 43 years, P2 aged 40 years, both due to sudden cardiac death.

### Whole exome sequencing

WES was performed on genomic DNA in P1 and P2 and on their mother. Their father was deceased at the time of study. The genomic DNA was exome-enriched using Illumina TruSeq 62 Mb exome capture and sequenced on an Illumina HiSeq 2000 with 100 bp paired-end reads. Standard filtering criteria were applied, including minor allele frequency of less than 1% and high-to-moderate variant effect predictor (i.e., nonsense, splice site, frame-shift, in-frame, and non-synonymous variants). Rare homozygous and compound heterozygous variants were defined, and protein altering and/or putative ‘disease causing’ mutations, along with their functional annotation, were identified using ANNOVAR. Candidate genes were prioritized if previously associated with a disease phenotype. Genomic and phenotypic data have been submitted to RD-Connect Genome-Phenome Platform (GPAP, https://platform.rd-connect.eu), where they can be accessed under a controlled access agreement.

### Detection of homozygous regions

As part of the RD-Connect GPAP standard analysis pipeline, runs of homozygosity (ROH) were identified in each individual using PLINK v.1.90 [6], applying optimized parameters as defined by Kancheva et al. [7]. Only ROH with a minimum length of 1 Mbp were identified, to exclude common shorter ROH. Consanguinity assessment was performed through application of ROH thresholds for WES as described in Matalonga et al. [8].

## Results

**Fig. 2:**
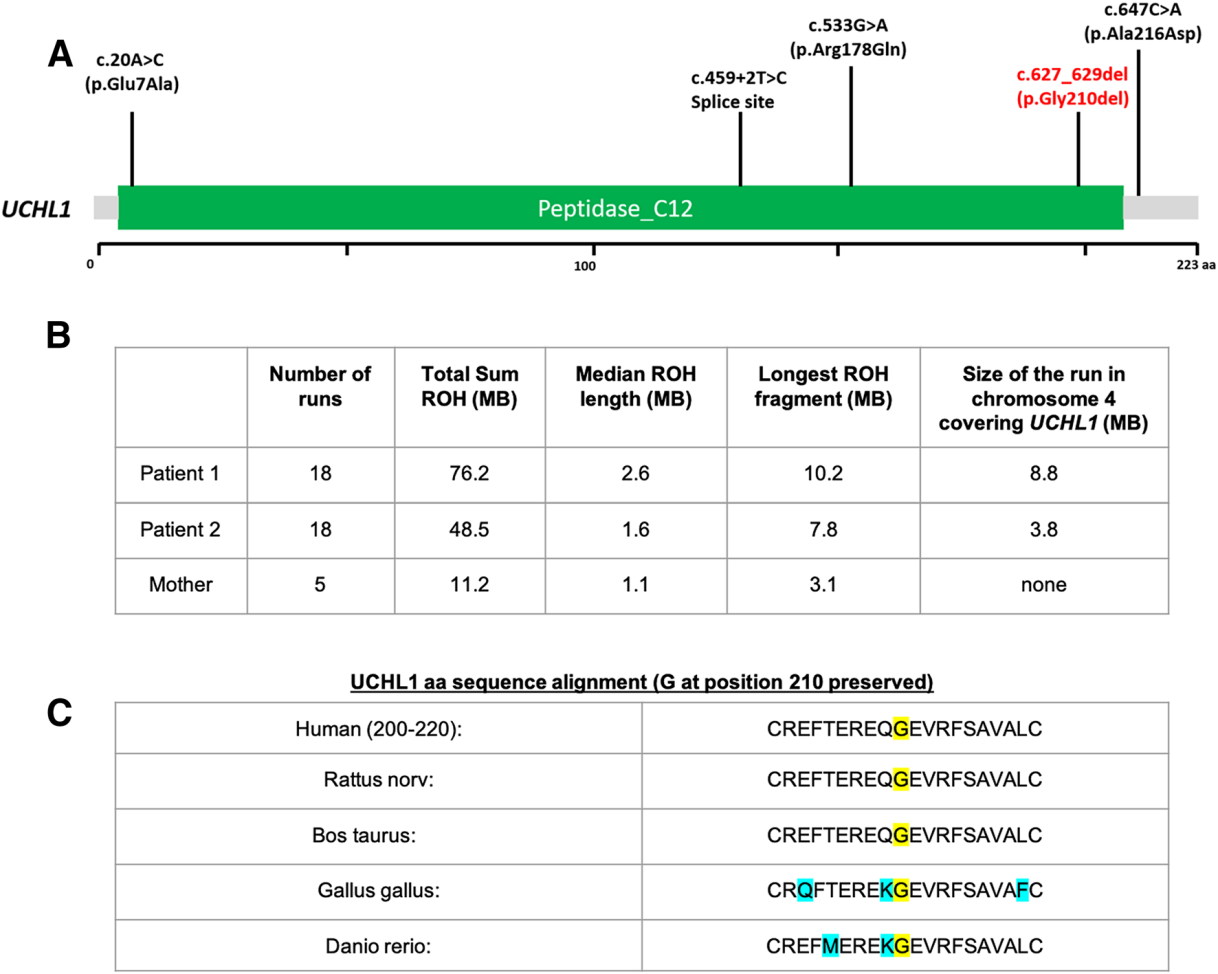
**a** Pathogenic mutations identified in *UCLH1*. **b** Consanguinity analysis through run of homozygosity (ROH) > 1 Mbp detection in autosomal chromosomes. **c** Conservation of the amino acid affected by the c.627_629del; p.(Gly210del) *UCHL1* mutation

WES analysis revealed the presence of a homozygous c.627_629del; p.(Gly210del) *UCHL1* variant (NM_004181.5) in P1 and P2 (Fig. 2a). This variant is reported only once in heterozygous form in gnomAD (allele frequency 0.000004), but never in homozygous form. Their unaffected mother was heterozygous for the variant. This variant could disrupt substrate binding at the distal UCHL1 site [9]. Additionally, we assessed IBD regions by the detection of > 1 Mbp runs of homozygosity (ROH). Results have shown that both patients carry an increased number of large ROH segments, 18 (P1 and P2) versus 5 from their mother, with a total ROH size of 76 MB (P1) and 48 MB (P2) respectively, underlying a possible recent consanguinity (Fig. 2b) [8]. Exome data were also analyzed for variants in genes associated with hypertrophic cardiomyopathy, but no potentially causative variants were detected.

## Discussion

Here, we describe two siblings with clinical presentation comprising progressive spasticity, motor neuropathy, ataxia, and optic atrophy, resembling Behr syndrome, and with the additional clinical finding of hypertrophic cardiomyopathy. Whole exome sequencing identified a novel homozygous deletion of three nucleotides in *UCHL1*. Paternal data were not available to perform complete segregation studies and confirm his carrier status. However, normal CGH array results excluded a large chromosomal rearrangement in the paternal allele, and although a deletion in *UCHL1* cannot be discarded, the possible recent consanguinity identified by ROH analyses support the homozygous status assumption. Ubiquitin C‐terminal hydrolase‐L1 (UCHL1) is critically important in maintaining free ubiquitin levels through the addition or removal of ubiquitin to poly-ubiquitin chains, and thus has a central role in cytoplasmic protein degradation [10]. It is one of the most abundant proteins in the nervous system, being highly expressed in both the central and peripheral nervous system [11–13]. Through its close interaction with the neuronal cytoskeleton, UCHL1 is thought to have important roles in axonal repair after injury, axonal transport, and synaptic function [14–16].

Mutations in *UCHL1* have been described in three families with autosomal recessive spastic paraplegia (SPG79) [3–5]. The phenotype of the patients in the present study had many similarities to the previously reported cases (Table 1). In all cases described to date, the first symptom was of childhood-onset visual loss due to optic atrophy. The subsequent disease course was characterized by varying degrees of spasticity and cerebellar ataxia. Additional, less consistently described features include epilepsy, cognitive impairment, facial myokymia, fasciculations, and reduced peripheral sensation, reflecting the widespread expression of *UCHL1* in most neuronal cells. The clinical presentation of our patients and all previously reported cases were compatible with Behr syndrome (Table 1).

**Table 1:** Clinical and molecular findings in patients with SPG79 in the patients described in this study and in the patients described by Bliguvar et al. [4], Rydning et al. [5], and Bhowmik et al. [3]

Paper	Bilguvar et al. [4]			Rydning et al. [5]			Bhowmik et al. [3]		Present family	
Patient	NG 1024–1	NG 1024–2	NG 1024–3	Patient III-4	Patient III-5	Patient III-6	P1	P2	P1	P2
Age at examination	28 y	33 y	34 y	65 y	62 y	62 y	10 y	7 y	41 y (died at 43)	33 y (died at 40)
Age at onset	5 y	7 y	5 y	10 y	10 y	10 y	2 y	2 y	8 y	8 y
First symptom	Visual loss	Visual loss	Gait imbalance	Visual loss	Visual loss	Visual loss	Delayed development and seizures	Delayed development and abnormal gait	Visual loss	Visual loss
Optic atrophy	+	+	+	+	+	+	+	+	+	+
Nystagmus	+	+	+	+	+	+	+	+	−	+
Ophthalmoparesis	NA	NA	NA	−	+	−	−	−	−	+
Pyramidal signs	+	+	+	+	+	+	+	+	+	+
Spasticity	+	+	+	−	+	+	+	+	+	+
Peripheral motor neuropathy	+	+	+	+	+	+	+	+	+	+
Pes cavus	NA	NA	NA	+	+	+	−	−	+	+
Sensory loss	+	+	+	+	+	+	−	−	−	−
Cerebellar signs	+	+	+	+	+	+	+	+	+	+
Lost ambulation	+	+	+	+	+	+	+	+	+	+
Seizures	−	−	+	−	−	−	+	−	−	−
Cognitive impairment	NA	+	+	−	−	−	+	+	−	−
Facial dysmorphism	NA	NA	NA	NA	NA	NA	+	+	+	+
Chest deformity	NA	NA	NA	−	+	+	+	+	+ (Scoliosis)	+ (Scoliosis)
Mutations reported	c.20A > C (p.Glu7Ala) homozygous			c.533G > A (p.Arg178Gln); c.647C > A (p.Ala216Asp) compound heterozygous			c.459 + 2 T > C (homozygous)		c.627_629del (homozygous)	

*y* years, *NA* not available, + present,− absent

First described by Carl Behr in 1909, the classical Behr syndrome (MIM 210000) comprises childhood-onset optic atrophy combined with various neurological symptoms including spastic paraparesis, ataxia, mild ophthalmoparesis, peripheral neuropathy, and a variable degree of learning difficulties [17]. While the optic atrophy in these patients remains stable, other neurological signs progress during childhood becoming more prominent in the second or third decades [17].

Autosomal recessive *OPA3* mutations have been identified in Iraqi Jewish patients presenting with Behr syndrome and 3-methylglutaconic aciduria (Costeff syndrome) [18] and more recently autosomal dominant [19] and recessive [20] *OPA1* mutations were shown in Behr syndrome without metabolic abnormalities. We reported six patients with Behr syndrome and autosomal recessive mutation in *C12orf65* [21], and a homozygous *C12orf19* mutation has also been reported to cause this phenotype [22], illustrating further genetic heterogeneity of this phenotype. All previously reported genetic forms of Behr syndrome affect mitochondrial proteins involved in different mitochondrial functions. The detection of *UCHL1* mutations in this phenotype adds another molecular mechanism, protein de-ubiquitination contributing to Behr syndrome and raising the possibility that this gene is also contributing to mitochondrial protein quality control.

In contrast to previously described cases, however, the siblings in this study both presented with the additional clinical feature of hypertrophic cardiomyopathy. Specifically, both patients demonstrated globally hypertrophied left ventricles in the absence of acquired causes of ventricular dysfunction (e.g., hypertension, myocardial ischaemia, valve dysfunction, prior exposure to toxins, and environmental pathogens). In addition, detailed family history failed to identify other family members known or suspected to be affected by a myocardial disease. The tissue distribution of UCHL1 is predominantly within the nervous system, although it is also expressed at low levels in gonadal cells [23]. Interestingly, UCHL1 is also upregulated in other cells under specialized conditions, for example in fibroblasts during wound healing and in pancreatic, colorectal, medullary thyroid, and breast cancer cells [24–27]. In these conditions, it is suggested that deregulation of the ubiquitin–proteasome system (UPS) may lead to uncontrolled cell growth. Furthermore, the UPS plays a key role in maintaining protein homeostasis and cardiac function, and UCHL1 has been shown to play a key role in this regulation. Knockdown of UCHL1 in cardiomyocytes and mouse hearts rescued cardiac hypertrophy induced by agonist or pressure overload, and overexpression resulted in the opposite effect [28]. We have not carried out functional studies of mutated UCHL1 protein (p.Gly210del). The Glycine residue at position 210 is close to the C-terminus, which regulates protein aggregation and stability [29]. The removal of the final four amino acids from the C-terminus renders the protein insoluble and abolishes binding to ubiquitin substrates [30]. Glycine at position 210 is located just upstream of the final beta-sheet and may have an impact on protein folding and stability. Further studies will be required to ascertain how the c.627_629del; p.(Gly210del) *UCHL1* mutation might lead to clinical hypertrophic cardiomyopathy. We recognize that it is not possible to completely exclude an alternative genetic or acquired cause for hypertrophic cardiomyopathy in these siblings, or an unrecognized cardiac disease in previous family members. Nonetheless, the presentation in these cases raises the possibility that hypertrophic cardiomyopathy may be an additional feature of the clinical syndrome associated with *UCHL1* mutations. The sudden cardiac death in both patients highlights the importance of cardiac follow-up and treatment in neurodegenerative disease associated with *UCHL1* mutations.

In summary, we have reported on a novel *UCHL1* deletion, resulting in a progressive neurodegenerative syndrome. The additional feature of hypertrophic cardiomyopathy has not been previously described. Cardiac evaluation is, therefore, essential in cases with this rare neurological phenotype.

## Data Availability

Whole exome sequencing data of the patients and their mother have been uploaded to the RD-CONNECT platform for data sharing and storage.
